# Psychometric investigation of the gamification Hexad user types scale in Brazilian Portuguese

**DOI:** 10.1038/s41598-022-08820-x

**Published:** 2022-03-22

**Authors:** Ana Cláudia Guimarães Santos, Wilk Oliveira, Maximilian Altmeyer, Juho Hamari, Seiji Isotani

**Affiliations:** 1 Institute of Mathematics and Computer Science, University of São Paulo, São Carlos, Brazil; 2 Gamification Group, Faculty of Information Technology and Communication Sciences, Tampere University, Tampere, Finland; 3 German Research Center for Artificial Intelligence, Saarbrücken, Germany

**Keywords:** Computer science, Information technology

## Abstract

Gamification has become a significant direction in designing technologies, services, products, organizational structures, and any human activities towards being more game-like and consequently being more engaging and motivating. Albeit its success, research indicates that personal differences exist with regards to susceptibility to gamification at large as well as to different types of gamification designs. As a response, models and measurement instruments of user types when it comes to gamification have been developed. One of the most discussed related instruments is the Hexad user types scale. However, there has been paucity of research related to the validity and reliability of the Hexad instrument in general but also of its different formulations and language versions. To face this gap, our study focused on analyzing the psychometric properties of the Hexad scale in Brazilian Portuguese by conducting two confirmatory factor analyses and two multi-group confirmatory factor analyses. The survey was answered by 421 Brazilian respondents (52% self-reported women, 47% self-reported men, 0.5% preferred not to provide their gender, and 0.5% checked the option “other”), from the five Brazilian regions (23 different states and the Federal District), and aged between 10 and 60 years old. Findings support the structural validity of the scale as an oblique model and indicate opportunities for small improvements. Further research, both at academy and practice, may use this study as the source of measurement of user types related to gamification (in Brazilian Portuguese), as well as, as a theoretical and practical source for further studies discussing personalized gamification.

## Introduction

Gamification refers to transforming systems, services, and activities to better afford similar motivational benefits as games often do^1^. It has been used across a wide range of human activities ranging from education to well-being^2–4^, to improve users’ experience and engagement^4^. After its introduction as an own research field roughly a decade ago, researchers primarily investigated *whether* gamification works^5^. Although these investigations showed that gamification leads to positive outcomes in most cases, also neutral or even negative outcomes have been reported^3,5,6^. This prompted gamification research to focus on understanding *how* and *why* it works (or not)^2^. It was found that interpersonal differences exist in the perception of gamification elements, making personalization, i.e. adapting gamified systems to the individual user, an important topic in the field^4^.

Past research has shown that personality traits or demographic factors like age or gender play a role in how gamification elements are perceived^4^. However, none of the aforementioned factors are particularly suitable for personalizing gamified systems, which is why there was a need for a dedicated theoretical model focusing on explaining inter-personal gamification preferences. The Hexad user types model, which was introduced by Marczewski^7^, satisfies this need. The Hexad is, as far we know, the only available user trait model which was specifically developed for the context of gamification (rather than games)^8^ and has been empirically validated by Tondello *et al*. in 2019^9^. Despite being recent, the Hexad has been used widely to tailor gamified systems to the user^4^ and has been shown to be superior in explaining user preferences for gamified systems compared to personality traits and other player typologies^10^. The model is based on Self-Determination Theory^11^, a major theory of human motivation, and consists of six user types which differ in the degree to which they are driven by intrinsic and extrinsic motivations: *Achievers* (motivated by mastery), *Players* (driven by extrinsic rewards), *Socialisers* (appreciating social interaction), *Philanthropists* (motivated by purpose), *Free-Spirits* (driven by exploration) and *Disruptors* (who like to trigger change).

The Hexad user types scale (composed of 24 non-invasive items) to access these user types was initially created in English^12^, and since its development, has been validated in different languages. Akgün and Topal^13^ conducted a study where they adapted this first version of the scale^12^ into Turkish. After presenting weak load values and a high error rate in the confirmatory factor analysis (CFA), two items (one from Player and another from Disruptor sub-scale) were removed from the final scale in Turkish. The study conducted by Tondello *et al*.^9^ was the first study validating the scale in English and Spanish, presenting new items and also indicating the necessity to validate this new version in other languages. Taşkın and Çakmak^14^ using the Single-Translation Method and focusing on the meaning rather than verbatim, conducted a study to adapt this new validated scale^9^ into Turkish. More recently, Krath and von Korflesch^15^ conducted a study to investigate the relationship between user types and game elements preferences, where they also conducted a validation of the Hexad scale in English and German. They indicated that even though the instrument was adequate in both language validations to identify the user types, both scales needed improvements to achieve a better model fit.

Also, two different studies focused on the validation of the scale specifically to adolescents, Ooge *et al*.^16^ conducted a validation process of the Hexad scale in Dutch and Manzano-León *et al*.^17^ in Spanish. In the validation in Dutch with adolescents, Ooge *et al*.^16^ were not able to confirm the validity of the scale, showing that the scale may not be suitable for adolescents. They proposed a further investigation about the scale and also a simplification of some items, becoming closer to the adolescents’ language. On the other hand, the study conducted by Manzano-León *et al*.^17^ was able to validate the scale in Spanish with adolescents, and also has shown that the instrument can be used regardless of gender (*i.e.*, boys and girls understood the scale in the same way).

Although these validation studies provided different versions of the scale, missing validated translations of the instrument prevent its use among other non-native speakers. As a consequence, albeit its proven scientific and practical value, researchers and practitioners in many countries cannot make use of the Hexad scale. In the study to validate the scale in English and Spanish, Tondello *et al*.^9^ tried to validate the scale in Brazilian Portuguese, however, they did not collect enough answers to conduct statistical analyses. In this article, we contribute to this issue by analyzing the psychometric properties of the Hexad scale in Brazilian Portuguese, a language spoken by more than 211 million native speakers, of which only 5,1% of the population have good English comprehension skills^18^. Consequently, we enable researchers to both recruit from a larger and thus potentially more representative pool of participants as well as increase the scientific validity of studies incorporating the Hexad scale in this language.

As far as we know, our study is the first to analyze the psychometric properties of the Hexad scale^9^ in Brazilian Portuguese. We report findings from an online study with 421 participants, in which we analyzed the reliability and validity of the translated instrument, by conducting two CFA. We also conducted two Multi-group Confirmatory Factor Analysis (MGCFA) to confirm that men and women understood the instrument in the same way, and analyzed the correlations between the Hexad user types. Our results support the structural validity of the translated scale as an oblique model (*i.e.*, with correlations between sub-scales) and indicate that 22 of the 24 items have an acceptable internal consistency. We also identified that there are opportunities for improvement in the future.

## Method

In this study, our goal was to analyze the psychometric properties of the gamification Hexad scale proposed by Tondello *et al*.^9^ (originally in English and Spanish) in Brazilian Portuguese. Initially, the survey was presented to the respondents as an online survey through the platform Google Forms, consisting of two sections: (i) demographic data (age, gender (male, female, other, and I prefer to not inform), educational level, state (Brazil has five large geographic regions, with 26 states and one Federal District)), and gaming habits (if the respondent play games and the frequency (every day, every week, rarely, and I do not know)), and (ii) the Hexad scale (composed of 24 statements, four items for each sub-scale). The Hexad scale items were presented on a 7-point Likert scale^19^, as recommended in the original study^9^. To avoid responses from people who did not pay due attention when reading and answering the statements, following other studies in the area^8,10^, we inserted an “attention-check” statement (*i.e.*, “I like to be with my friends, but this question is just to evaluate your attention. Please, mark option number 3, to let us know that you are paying attention”). This “attention-check” statement was in the middle of section two (i.e., the Hexad scale), and similar to the Hexad items, was presented on a 7-point Likert scale. Responses from people who missed the attention-check statement were removed from the data analysis. In addition, the 24 items of the Hexad scale were presented to participants in a random order, as recommended by Tondello *et al*.^12^.

The 24 items of the Hexad scale were the items available (in Portuguese) on the website of the HCI Games Group. According to Tondello *et al*.^9^, two independent native speakers separately translated all the statements and descriptions into Brazilian Portuguese from the original version. Besides, each item was compared and assessed by an independent third native speaker. The original scale (in English) and the scale used in the study, can be seen in supplementary Table S1. After the survey construction and before the official survey release, as recommended by Connelly^20^, two researchers conducted a pilot study by applying the survey to 10 people where they evaluated the size of the survey. The participation in this pilot study was voluntary, the respondents also had to pass in the “attention-check” statement, and eight of the ten participants evaluated the survey size as adequate.

### Participants

Two researchers conducted the data collection. Aiming to have participants with different backgrounds, participants were recruited via email lists (academic and non-academic) and social networks (Facebook, Instagram, and Twitter) between March and October of 2020. The email lists were from personal contacts of the researchers, from participants of previous researches and also from participants that made available their emails in a conference of educational technology organized by one of the researchers. The propagation through Twitter, Facebook, and Instagram was made in the researchers’ personal accounts, and in the propagation in Facebook, the researchers also posted about the research in public groups about gamification. All the postings in social networks were not targeted at any kind of ads and the publications were made public to facilitate the propagation by others. Considering that volunteers might be more willing to pay attention in surveys and also without pressuring to maximize time usage^21^, participation in the study was entirely voluntary. Participants had to accept to participate by checking a consent term, where they were informed about the purpose of the study, the study confidentiality, that the data collected would be used in scientific studies, and also the contact of the researchers and universities involved in the study. Similar to the original study, participants could quit the study at any time before submitting responses.

463 responses were collected, of which 42 were discarded for having missed the attention-check item. With that, we analyzed data from 421 participants (219 women, 198 men, two participants preferred not to provide their gender, and two reported themselves as “other”). We received responses from 23 Brazilian states and the Federal District, covering the five geographic regions of Brazil. Besides, the number of participants in our study (N = 421) can be considered acceptable for CFA and multi-group CFA (MGCFA) by gender, since different authors have indicated as recommendations regarding the minimum necessary sample size in factor analysis a sample of at least 100, with a sample of 200 being considered fair, and a sample of 300 being considered a good sample^22,23^. Most of the respondents had at least a bachelor degree (Elementary/Middle/High School = 10%; Bachelor = 32%; Specialized/MBA Courses = 21%; M.Sc. = 24%; PhD = 13%), and were older than 20 years (10 to 19 = 9%; 20 to 29 = 29%; 30 to 39 = 28%; 40 to 49 = 23%; 50 to 59 = 10%; over 60 = 1%). Therefore, we were able to collect data from people with different demographic backgrounds. Also, most of the respondents (68%) reported that playing games were a habit.

### Statistical analysis

We analyzed the (i) descriptive statistics, (ii) internal reliability, (iii) correlation between user types, and (iv) factor analysis of the data. As the aim of the study was to assess the psychometric properties of a model (Hexad scale), according to Levine^24^ a CFA is a more appropriate procedure in comparison with an EFA. Similar to Manzano-León *et al*.^17^, we also conducted two MGCFA aiming to analyze gender invariance (*i.e.*, to assess whether the survey is understood in the same way by men and women).

The data were analyzed using IBM SPSS 27^25^ and JASP 0.14.1^26^. We used the software IBM SPSS 27^25^ to conduct a Kolmogorov-Smirnov test (aiming to see if the data was parametric or non-parametric), to measure the descriptive statistics (mean, the standard deviation, and the data variances in each sub-scale), the internal reliability (Cronbach’s $\alpha$), and the bivariate correlation coefficients (Kendall’s $\tau$) in the data obtained. We also used this software to conduct a Wilcoxon test^27^, to see if there was a significant difference between the answers based on the genders and a Friedmans test^28^ with Bonferroni adjustment to test the difference between the user types.

We used the software JASP 0.14.1^26^ to conduct the CFA, using structural equation modeling (SEM) with a robust maximum likelihood method. Considering that the Kolmogorov-Smirnov test showed that our data were non-parametric, we used the robust option of the method as it is more suitable for analyzing data that does not follow a normal distribution^29^. To assess the model fit, we analyzed the Chi-Square ($\chi ^2$), the Relative Chi-square ($\chi ^2/df$), the Goodness of Fit Index (GFI), the Tucker-Lewis Index (TLI), the Comparative Fit Index (CFI), the Bentler-Bonett Normed Fit Index (NFI), the Standardized Root Mean Square Residuals (SRMR) and the Root Mean Square Error of Approximation (RMSEA) results. Based on different studies’ recommendations^30–34^ we considered the goodness-of-fit indexes as $\chi ^2$ p $\ge$ 0.05; $\chi ^2/df$ $\le$ 3; GFI $\ge$ 0.95; TLI $\ge$ 0.95; CFI $\ge$ 0.95; NFI $\ge$ 0.95; SRMR $\le$ 0.08; and RMSEA $\le$ 0.06.

Since prior research about the Hexad scale conducted different CFA (considering an orthogonal model or an oblique model), we conducted two different CFA. In the first one, the factors were not correlated, therefore, the six Hexad user types were modeled as latent variables, the 24 survey items were modeled as observed variables, and the four items associated with each user type modeled as reflections of the respective latent variable. The second CFA was conducted correlating the factors, with the six Hexad user types modeled as latent variables correlated with each other, the 24 survey items modeled as observed variables, and the four items associated with each user type modeled as reflections of the respective latent variable.

We carried out two MGCFA (one considering an orthogonal model and another considering an oblique model), to confirm whether the factor structure of the scale is invariant according to the gender of the respondent (i.e. women and men understood the scale in the same way). In this analysis, we only considered the data from respondents that self-reported their gender as male or female (417 answers from the 421 answers collected). The analysis was carried out in the software JASP 0.14.1^26^, using the robust maximum likelihood method, and evaluating the invariance of three models (unconstrained, metric, and scalar). The first model (unconstrained model) evaluated whether the number of items and factors were acceptable for both genders, the second model (metric invariance) analyzed whether the factor loadings of the items could be considered equivalent between the genders, and the third model (scalar invariance) investigated whether the level of latent trait needed to endorse the item categories (thresholds) was equivalent between the genders. To the evaluation of the model, we considered as goodness-of-fit indexes: RMSEA $\le$ 0.06, SRMR $\le$ 0.08, CFI $\ge$ 0.95, and TLI $\ge$ 0.95^30,35^. The invariance was evaluated using the CFI difference test ($\Delta$CFI). When the difference between the $\Delta$CFI of the models is under 0.01, the results indicate the invariance of the model^36^.

## Results

In this section, we present the results from the analyses of internal reliability, distribution of the Hexad user types, correlations presented between the user types, and the results from the confirmatory factor analyses and gender invariance.

### Internal reliability, correlations and user type distribution

Initially, we analyzed the distributions of the responses for all variables by using Kolmogorov-Smirnov test^37^, which results showed that the scores from all the variables were not normally distributed. We also measured the descriptive statistics (Mean, the standard deviation, and the data variances in each sub-scale), the internal reliability analyses (Cronbach’s $\alpha$), as well as the bivariate correlation coefficients (using Kendall’s $\tau$). Supplementary Table S2 presents the results. Considering that each Hexad sub-scale has four items rated on a 7 point Likert-scale, the minimum value a sub-scale can be is 4 and the maximum value a sub-scale can be is 28. Overall, the reliability scores are acceptable ($\alpha$ $\ge$ 0.70), except for the Disruptor sub-scale. Prior studies^9,16^ have also found similar results ($\alpha$ $\le$ 0.70) for the Disruptor sub-scale. Since the user type scores were non-parametric, as recommended by Wohlin *et al*.^38^, we measured the bivariate correlation coefficients between each Hexad user type using Kendall’s $\tau$. In our study, similar to Tondello *et al*.^9^, we identified a partial overlap between the user types, however in different levels.

As reported in supplementary Table S2, the higher average scores were from Philanthropists, Achievers, and Free Spirits, and the lower average scores were from Disruptors. These values are similar to other recent studies about the Hexad user types^9,14,17,39^. We also calculated the dominant user types (*i.e.* the strongest tendency of the respondents^10,40^), which distribution results were: Philanthropist = 34%, Achiever = 30%, Free Spirit = 13%, Player = 12%, Socialiser = 11%, and Disruptor = 1%. Philanthropists, Achievers, and Free Spirits were the dominant user types of 77% of the respondents, which were expected considering that the respondents presented higher average scores in these three user types. When considering gender, the distribution of the participants who self-reported as female was: Philanthropist = 35%, Achiever = 26%, Free Spirit = 14%, Player = 10%, Socialiser = 13%, and Disruptor = 1%, while the distribution of the participants who self-reported as male was Philanthropist = 32%, Achiever = 35%, Free Spirit = 10%, Player = 14%, Socialiser = 8%, and Disruptor = 1%. Therefore, there were more Philanthropists, Free Spirits, and Socialisers between the self-reported women, while the self-reported men presented a higher percentage of Achievers and Players as dominant user types.

To analyze if there was a significant difference in the user types according to their gender, as our data are non-parametric and the variables (users types) are related, following Wohlin’s *et al*.^38^ orientations, we conducted the Wilcoxon test^27^. The results show that there was no significant difference between the genders. We also tested the difference between the groups (user types). As our data are non-parametric and the variables are related, following Wohlin’s *et al*.^38^ recommendations, we conducted the Friedmans test^28^ with Bonferroni adjustment, thus, reducing the chance of Type-I errors^41^. The overall results ($\chi ^2_{5}$ = 915.834, *p* $\le$ 0.000) demonstrated a significant difference between the groups. The adjusted results indicate a difference between all of the groups, with an exception for Socialisers and Players, and Achievers and Philanthropists.

### Confirmatory factor analysis

**Figure 1:**
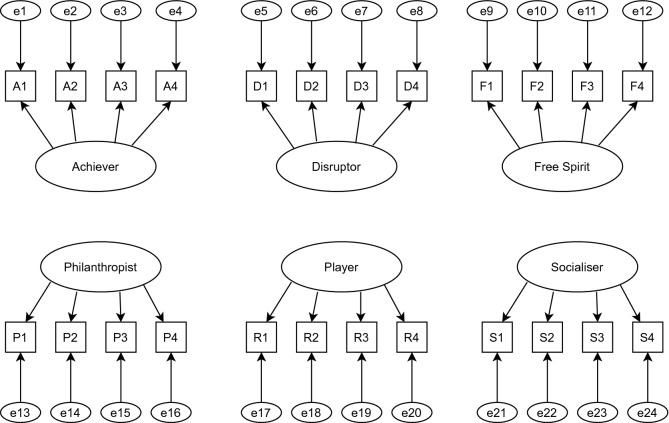
Path model (adapted from Tondello *et al*.^9^). The ellipses represent the factors and the rectangles represent the items of the scale.

When analyzing the path models of the studies that tried to validate the Hexad scale, it was possible to find two different approaches in the conduction of the CFA. While the studies conducted by Tondello *et al*.^9^ and by Ooge *et al*.^16^ considered the Hexad as an orthogonal model (*i.e.*, the six user types as factors without correlation between them), Akgün and Topal^13^, Taşkın and Çakmak^14^, and Manzano-León *et al*.^17^ considered the Hexad model as an oblique model (*i.e.*, the six user types as factors correlated to each other). Initially, we decided to replicate the CFA conducted by Tondello *et al*.^9^ and Ooge *et al*.^16^,i.e., considering the six user types as factors without correlation between them. Figure 1 presents the path model.

To evaluate the goodness of fit of the model, following Kline’s^42^ suggestion, we initially used the chi-squared test $\chi ^2$ and the root mean square error of approximation (RMSEA). The Chi-squared test did not support the evidence for a good model fit ($\chi ^2_{252}$ = 1910.204, *p* $\le$ 0.001). However, the Chi-squared test is sensitive to the sample size, normally rejecting the model fit when large samples are used and not discriminating good fitting models and poor fitting models when small samples are used^30^. Thus, we calculated the $\chi ^2/df$ = 3.6, which did not indicate a good model fit, however, indicated a fair fit^30,43^. The RMSEA = 0,125 (CI = [0.120, 0.130]) also did not support the evidences for a well-accepted fitted model^32^. The Comparative Fit Index (CFI) = 0.702, the Tucker-Lewis Index (TLI) = 0.673, the Bentler-Bonett Normed Fit Index (NFI) = 0.673, and the Standardized root mean square residual (SRMR) = 0.314 also did not indicate an acceptable fit of the model^30,32^. However, the Goodness of fit index (GFI) = 0.956, indicated a good fit^33^.

Table 1 present the factor loadings for each of the Hexad survey items in Brazilian Portuguese. Since Cronbach’s $\alpha$ may be misleading due to its tendency to underestimate reliability^44^, the composite reliability (CR) is a good option to measure the reliability considering that is formulated through structural equation modeling and is equivalent to coefficient omega^45^. Based on these factor loadings, we calculated the composite reliability (CR) finding acceptable values (CR $\ge$ 0.7) for all the factors except the Disruptor (Achiever = 0.874; Disruptor = 0.669; Free Spirit = 0.766; Philanthropist = 0.888; Player = 0.818; and Socialiser = 0.886).

**Table 1:** Factor loadings. *N* = 421. UT: User types/factors; I: Items; SE: standard errors; CR: critical ratios; CI: Confidence interval; $\lambda$: standardized $\lambda$; bold: $\lambda$ $\ge$ 0.500; A: Achiever; D: Disruptor; F: Free Spirit; P: Philanthropist; R: Player; S: Socialiser.

				**CI**		
UT	I	SE	Z-value	5%	95%	$\lambda$
**A**	**A1**	0.087	12.141	0.884	1.225	**0.836**
	**A2**	0.077	15.827	1.075	1.378	**0.790**
	**A3**	0.089	11.866	0.879	1.227	**0.779**
	**A4**	0.086	12.574	0.912	1.248	**0.779**
**D**	**D1**	0.105	9.822	0.825	1.237	**0.539**
	**D2**	0.109	7.890	0.645	1.072	0.491
	**D3**	0.106	11.758	1.035	1.449	**0.648**
	**D4**	0.099	11.950	0.985	1.371	**0.636**
**F**	**F1**	0.092	12.509	0.969	1.329	**0.793**
	**F2**	0.095	8.582	0.629	1.001	0.496
	**F3**	0.090	12.100	0.914	1.268	**0.807**
	**F4**	0.098	9.450	0.731	1.113	**0.560**
**P**	**P1**	0.081	13.543	0.940	1.258	**0.868**
	**P2**	0.073	16.172	1.042	1.329	**0.851**
	**P3**	0.083	13.072	0.926	1.252	**0.799**
	**P4**	0.089	11.585	0.856	1.205	**0.737**
**R**	**R1**	0.086	15.317	1.145	1.481	**0.694**
	**R2**	0.075	18.568	1.240	1.533	**0.855**
	**R3**	0.088	11.556	0.846	1.192	**0.640**
	**R4**	0.089	14.663	1.131	1.480	**0.712**
**S**	**S1**	0.069	19.810	1.224	1.493	**0.823**
	**S2**	0.063	23.211	1.332	1.577	**0.908**
	**S3**	0.075	16.355	1.087	1.382	**0.721**
	**S4**	0.068	19.214	1.165	1.430	**0.789**

In Table 2 we present the modification indices with values $\ge$ than 30.000. The modification index is an approximation of how much each parameter could decrease the $\chi ^2$ value, and therefore, improve the fit model, if freely estimated^35,42^. The expected parameter change (EPC) indicates an estimation of how much the parameter would change if freely estimated^35^. The results indicated that especially the item D2 has presented a correlation with all the factors, which indicates that possibly an improvement in this item would improve the model fit.

**Table 2:** Modification indices of the first CFA. *N* = 421.

	Modification Indices	Expected Parameter Change
Achiever $\rightarrow$ D2	111.430	0.884
Free Spirit $\rightarrow$ D2	98.619	0.864
Philanthropist $\rightarrow$ D2	88.796	0.781
Free Spirit $\rightarrow$ R3	76.296	0.613
Socialiser $\rightarrow$ D2	62.360	0.651
Socialiser $\rightarrow$ F4	49.211	0.512
Philanthropist $\rightarrow$ R3	47.072	0.458
Achiever $\rightarrow$ R3	46.979	0.463
Player $\rightarrow$ D2	37.588	0.524
Philanthropist $\rightarrow$ A1	35.505	0.252

In summary, when using a similar path model that Tondello *et al*.^9^ and Ooge *et al*.^16^ studies used, CFA demonstrated that the measurement model has not an acceptable fit considering our data, indicating that some items could be improved. One of the possible explanations for this result is that probably occurred an overlap of items measuring the same factor. The results demonstrated that items D2 and F2 were the weaker fit to their respective sub-scales (see Table 1).

**Figure 2:**
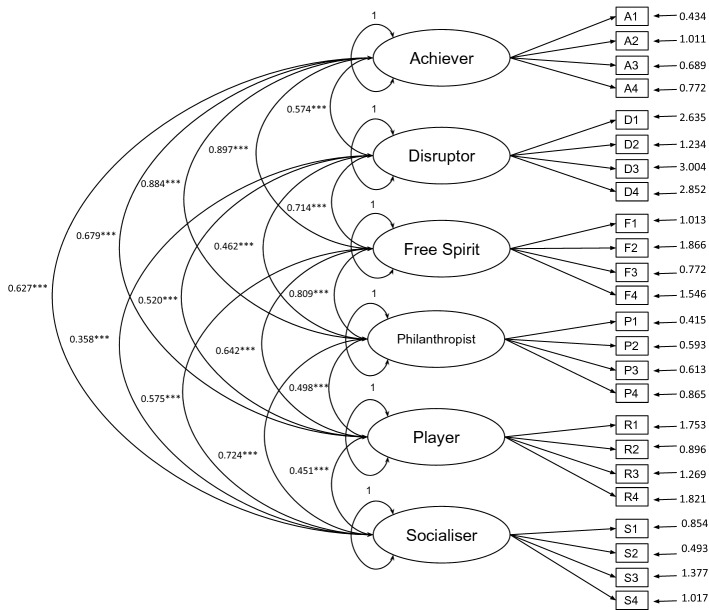
Path model with correlations between the factors. The ellipses represent the factors and the rectangles represent the items of the scale. *** $\mathrm{p} < 0.001$. The variance in each factor is defined in 1 by JASP^26^. All parameters were freely estimated in the analysis.

After analyzing the results from Kendall’s test (that indicated correlation between the user types) as well as the results from the first CFA (that indicated a poor fit model), we decided to conduct a second CFA considering the six factors as correlated to each other. This CFA replicated the analysis conducted by Akgün and Topal^13^, Taşn and Çakmak^14^, and Manzano-León *et al*.^17^. Figure 2 presents the path model.

The Chi-squared test did not support the evidence for a good model fit ($\chi ^2_{252}$ = 646.836, *p* $\le$ 0.001), however, differently from the first CFA, the $\chi ^2/df$ = 2.56, the RMSEA = 0.064 (CI = [0.058, 0.070]), and Standardized root mean square residual (SRMR) = 0.073, indicated a good fit^30^. The Comparative Fit Index (CFI) = 0.926, the Tucker-Lewis Index (TLI) = 0.914, the Bentler-Bonett Normed Fit Index (NFI) = 0.889, and the Goodness of fit index (GFI) = 0.882 were slightly below the acceptable values that would indicate a good fit^30,32^. Thus, even though some of the indices did not indicate the good fit of the model to our data, when considering the Hexad as an oblique model, the indices were closer to indicating a good fit model than in the first CFA. Table 3 presents the factor loadings from this second CFA. We also calculated the CR based on these factor loadings, finding acceptable values (CR $\ge$ 0.7) for all the factors except the Disruptor (Achiever = 0.873; Disruptor = 0.622; Free Spirit = 0.769; Philanthropist = 0.888; Player = 0.819; and Socialiser = 0.886).

**Table 3:** Second factor loadings. *N* = 421. UT: User types/factors; I: Items; SE: standard errors; CR: critical ratios; CI: Confidence interval; $\lambda$: standardized $\lambda$; bold: $\lambda$ $\ge$ 0.500; A: Achiever; D: Disruptor; F: Free Spirit; P: Philanthropist; R: Player; S: Socialiser.

				CI		
UT	I	SE	Z-value	5%	95%	$\lambda$
**A**	**A1**	0.081	13.273	0.918	1.235	**0.853**
	**A2**	0.076	15.548	1034	1.332	**0.762**
	**A3**	0.084	12.735	0.902	1.230	**0.789**
	**A4**	0.081	13.313	0.915	1.231	**0.774**
**D**	**D1**	0.090	11.228	0.835	1.188	**0.529**
	**D2**	0.094	14.431	1.169	1.537	**0.773**
	**D3**	0.107	7.602	0.606	1.027	0.426
	**D4**	0.105	7.252	0.554	0.965	0.410
**F**	**F1**	0.087	12.030	0.873	1.213	**0.719**
	**F2**	0.084	10.891	0.747	1.074	**0.555**
	**F3**	0.086	11.887	0.858	1.197	**0.760**
	**F4**	0.072	14.995	0.936	1.218	**0.655**
**P**	**P1**	0.082	13.322	0.929	1.249	**0.861**
	**P2**	0.072	16.070	1.020	1.303	**0.833**
	**P3**	0.080	13.969	0.959	1.272	**0.818**
	**P4**	0.086	12.099	0.875	1.213	**0.747**
**R**	**R1**	0.081	16.783	1.193	1.509	**0.714**
	**R2**	0.066	19.851	1.186	1.446	**0.812**
	**R3**	0.083	13.559	0.964	1.290	**0.707**
	**R4**	0.085	14.536	1.073	1.407	**0.677**
**S**	**S1**	0.067	20.386	1.238	1.501	**0.829**
	**S2**	0.061	23.679	1.321	1.560	**0.899**
	**S3**	0.074	16.921	1.102	1.390	**0.728**
	**S4**	0.067	19.376	1.167	1.429	**0.790**

In Table 4 we present the modification indices with values $\ge$ than 30.000. Again, the item D2 presented a correlation with most of the factors, however, in this analysis, the items D3 and D4 also presented some correlations with the factors. This might indicate the necessity of improvement in all the Disruptor sub-scale items.

**Table 4:** Second modification indices of the second CFA. *N* = 421.

	Modification indices	Expected parameter change
Free Spirit $\rightarrow$ D2	106.398	2.348
Achiever $\rightarrow$ D2	88.736	1.508
Philanthropist $\rightarrow$ D2	77.717	1.133
Free Spirit $\rightarrow$ R3	69.735	0.837
Achiever $\rightarrow$ R3	49.527	0.710
Socialiser $\rightarrow$ D2	43.896	0.738
Philanthropist $\rightarrow$ R3	41.049	0.515
Achiever $\rightarrow$ D4	39.436	$-$0.801
Philanthropist $\rightarrow$ D4	34.883	$-$0.655
Achiever $\rightarrow$ D3	32.154	$-$0.749
Free Spirit $\rightarrow$ D4	31.378	$-$0.928

When modeling the path model similar to Akgün and Topal^13^, Taşn and Çakmak^14^, and Manzano-León *et al*.^17^ studies, CFA results demonstrated that the model is closer to an acceptable fit but also can be improved. In this analysis, the items D3 and D4 were the weaker fit to the Disruptor sub-scale (see Table 3). After this CFA analysis and also considering the analysis made by Akgün and Topal^13^, Taşn and Çakmak^14^, and Manzano-León *et al*.^17^ studies, we understand that the Hexad is an oblique model, and that is why it presents a better fit model when correlating the items in the CFA.

### Gender invariance analysis

To measure the gender invariance, we carried out two MGCFA (one considering the orthogonal model and another considering the oblique model). In the first MGCFA (orthogonal model), the comparisons between the unconstrained model (RMSEA = 0.130 [0.124–0.135]; SRMR = 0.321; TLI = 0.655; CFI = 0.685), the metric invariance (RMSEA = 0.128 [0.122–0.133]; SRMR = 0.321; TLI = 0.666; CFI = 0.684; $\Delta$ CFI = −0.001), and the scalar invariance (RMSEA = 0.127 [0.121–0.132]; SRMR = 0.309; TLI = 0.671; CFI = 0.678; $\Delta$ CFI = −0.006), indicated an acceptable invariance ($\Delta$CFI $\le$ 0.01).

In the second MGCFA (oblique model), the comparisons between the unconstrained model (RMSEA = 0.123 [0.118–0.129]; SRMR = 0.097; TLI = 0.688; CFI = 0.715), the metric invariance (RMSEA = 0.121 [0.116–0.126]; SRMR = 0.103; TLI = 0.700; CFI = 0.684; $\Delta$ CFI = −0.001), and the scalar invariance (RMSEA = 0.120 [0.115–0.125]; SRMR = 0.101; TLI = 0.705; CFI = 0.706; $\Delta$ CFI = -0.008) also indicated an acceptable invariance ($\Delta$CFI $\le$ 0.01). Therefore, the results of both MGCFA demonstrated that the instrument in Brazilian Portuguese can be used regardless of gender, independent of model.

## Discussion

In this study, we focused on analyzing the psychometric properties of the Hexad user types scale^9^ in Brazilian Portuguese. To do so, we administered the so far non-validated Brazilian Portuguese version to 421 Brazilian respondents. Considering studies that validated the Hexad scale in other languages, we carried out reliability analysis, two different confirmatory factor analyses (CFA), and two multi-group confirmatory factor analyses (MGCFA) in our data set. Concerning the CFA, the CFA considering the Hexad an oblique model presented a closer good model fit, which might indicate that the best way to conduct CFA in the Hexad scale is assuming correlations between the six factors. However, both CFA indicated problems with the Disruptor sub-scale. The MGCFA indicated that the instrument can be used regardless of gender.

Considering the distribution of the scores, our results are similar to prior research^9,14,17,39^, demonstrating that Philanthropists, Achievers, and Free Spirits are the strongest tendencies of the users regarding the Hexad user types, while Disruptor is the lower tendency. We also calculated the dominant user types, indicating that Achiever and Philanthropist were responsible for more than 60% of the dominant user types of the respondents. Partially similar to the results found by Tondello *et al*.^9^, participants who self-reported as female, seemed to be more motivated by the Philanthropist, Free Spirit, and Socialiser tendencies while participants who self-reported as male, seemed to be more motivated by the Achiever and Player tendencies. The results from the study conducted by Oyibo *et al*.^46^, which indicated that male participants are more responsive to rewards strategies, might explain why they are more motivated by the Player user type.

Our results presented significant correlations between the user types, which some were expected taking into account that their underlying motivations are related^12^. The strongest correlation occurred between Philanthropists and Socialisers, and could be expected since both user types are interested in social interaction, with Socialisers interested in the interaction itself and Philanthropists interested in interaction for altruistic purposes^9^. These correlations between the user types might complicate the creation of items that only fit one user type, and also, we understand that these correlations between the user types are a theoretical indication that the Hexad is an oblique model.

In the first CFA, when not correlating the factors, our study did not present a good model fit ($\chi ^2/df$ = 3.6, RMSEA = 0,125,CFI = 0.702, TLI = 0.673, NFI = 0.673, and SRMR = 0.314), and also two items presented $\lambda$ $\le$ 0.5 (D2: $\lambda$ = 0.491, and F2: $\lambda$ = 0.496). In our study, similar to Tondello *et al*.^9^, the item F2 presented a low factor loading. Tondello *et al*.^9^ indicated this item as passive of improvement, suggesting that it would probably fit better with another user type. Considering the problems that other studies presented with the Free Spirit sub-scale, and that the item F2 might be related with other user types^9,16^, it is important to conduct future studies improving the sub-scale (specifically the item F2). Regarding the item D2, we think a possible problem with the item is the use of the Latin expression “status quo”. In other validation studies, this expression was replaced by another expression in the validation language^9,14^, or the respondents were informed about the meaning of the expression^16,17^. Therefore, we think that a reformulation of this item or a previous explanation about the expression “status quo” to the respondents, could improve the understanding and consequently, the results. In the CFA correlating the factors, the fit indices were acceptable or close to acceptable ($\chi ^2/df$ = 2.56, RMSEA = 0.064, SRMR = 0.073, CFI = 0.926, TLI = 0.914, NFI = 0.889, and GFI = 0.882). Overall, the Disruptor sub-scale presented some problems (*i.e.*, items with $\lambda$ $\le$ 0.5, Composite Reliability below the acceptable, and items presenting modification indices with values $\ge$ than 30.000), which we understand as an indication that a further investigation about this user type might be necessary to learn more about how people present its characteristics.

Similar to Manzano-León *et al*.^17^, we also conducted MGCFA to assess whether gender could influence the understanding of the scale. Since we conducted two CFA, we decided to conduct two MGCFA (one correlating the factors and another not correlating the factors) to test the invariance of the scale. In both MGCFA, the invariance was evaluated using the CFI difference test ($\Delta$CFI $\le$ 0.01) indicating unconstrained, metric, and scalar invariance^36^. Albeit prior research has investigated how gender could affect the distribution of the Hexad user types^9,47^, less is known about whether the same scale can be used for women and men. Analysis of how women and men understand the Hexad scale is important considering that gender can play an important role in gamification design^48,49^. Prior research has indicated that the preference for game elements can change depending on the gender^50–52^, therefore, it is important to analyze if gender also has influence when defining the user type through a scale. Based on our results and the results presented by Manzano-León *et al*.^17^, the Hexad scale is an instrument that can be used regardless of gender.

Overall, the different analyses conducted in this study demonstrated that the Brazilian Portuguese version of the Hexad scale is an instrument that is near to complete validation and can be used regardless of gender. The scale evaluated in this study can be used to identify the Hexad user types in future research involving Brazilian samples, at the same time that practitioners can use our results as a guide to modeling gamified systems according to the Hexad user types. The use of this translated instrument can be an effective option for researchers and practitioners to group people into different user types in a gamified context, and therefore, personalize gamified systems or conduct further analysis about user behavior and motivations on this type of system.

## Limitations and opportunities for the future

This study has presented some limitations concerning different aspects. Considering the demographic information of the respondents, we were not able to collect answers in all Brazilian states, and also some regions had low participation, which prevented us to present possible correlations between the user types and demographic characteristics. Considering the age of the respondents, most of them were older than 20 years, therefore, the results here presented might not be applicable to children and teenagers. We analyzed the psychometric properties of the Hexad scale translated to Brazilian Portuguese, however, other countries also have Portuguese as the official language (*e.g.* Portugal, Angola, Mozambique), and the instrument used in this study might not be the most suitable to be used in these countries.

Based on these limitations, we propose some studies that can be carried out in the future. (i) Following other studies that tried to validate the scale for young people^16,17^, we propose future studies specifically to analyze the psychometric properties the Brazilian Portuguese scale for adolescents. This validation with younger people can help designers to personalize gamified settings specifically developed for them (e.g. educational gamified environments for adolescents). (ii) Since there were items that did not reach the expected factor loading values in this study, future studies can propose new translations for them as well as new items to measure the Disruptor and Free Spirit sub-scale. These improvements can increase the reliability in these sub-scales and also the power of these items in the measurement of the Hexad user types. (iii) Finally, considering that the countries that have Portuguese as the official language present cultural differences and also some differences in the language itself, future studies can adapt the Brazilian Portuguese scale to other Portuguese-speaking countries, making possible the use of the scale in more locations.

## Conclusion

Having models that identify the user types in gamified settings is a current challenge. Although there is already a scale to measure the users’ profile considering gamification aspects (*i.e.*, Hexad), this has not yet been validated in several widely spoken languages (*e.g.*, Brazilian Portuguese), failing to benefit a large number of researchers and practitioners. In this study, we analyzed the psychometric properties of the Hexad scale in Brazilian Portuguese. Our results demonstrated that the Brazilian Portuguese version has good internal reliability, CFA values acceptable or near to the acceptable (needing special attention the Disruptor sub-scale), and that there were overlaps between the user types. These overlaps between the user types as well as the statistical results when modeling the CFA with correlated factors indicate that the best way to conduct CFA of the Hexad scale in the future is considering the Hexad as an oblique model. The study results indicated that the model is close to complete validation, but some items still need to be improved. As future studies, we intend to analyze and adapt the items that presented a low factor loading and then replicate the study with new participants. We also aim to adapt and analyze the psychometric properties of the scale in Portuguese from other Portuguese-speaking countries. Other studies improving the scale could help a considerable number of researchers that conduct studies with Brazilian respondents. Having a validated Hexad scale in Brazilian Portuguese can represent a significant advance in identifying the profile of the users and, consequently, in the personalization of several types of gamified systems.

### Ethical statements

This study has been performed in accordance to the Brazilian National Health Council resolution number 510 published on April 7th, 2016, and with the relevant guidelines and regulations set by the Universities involved. Informed consent for participation was obtained from all participants.

## Supplementary Information

Supplementary Information 1.

Supplementary Information 2.

Supplementary Information 3.

## Data Availability

The dataset generated and analyzed during the current study is available as supplementary material.
