# Supplementary material for: Psychometric investigation of the gamification Hexad user types scale in Brazilian Portuguese
Source: Sci Rep. 2022 Mar 22;12:4920. doi: 10.1038/s41598-022-08820-x (PMC8940910; doi:10.1038/s41598-022-08820-x)
Supplement: Supplementary file 3 — Supplementary Information 3. [file 41598_2022_8820_MOESM3_ESM.pdf]

UT	M	SD	Var	α	A	*P-value*	D	*P-value*	F	*P-value*	P	*P-value*	R	*P-value*
**A**	23.90	4.733	22.401	0.871										
**D**	14.77	5.274	27.811	0.669	0.186**	0.000								
**F**	22.52	4.614	21.288	0.748	0.424**	0.000	0.310**	0.000						
**P**	24.18	4.681	21.909	0.885	0.464**	0.000	0.099**	0.005	0.372**	0.000				
**R**	20.63	5.572	31.052	0.812	0.377**	0.000	0.229**	0.000	0.337**	0.000	0.216**	0.000		
**S**	20.57	5.690	32.378	0.882	0.338**	0.000	0.087*	0.012	0.300**	0.000	0.483**	0.000	0.271**	0.000

Table S2. Descriptive analysis, internal reliability, and bivariate correlation coefficients (Kendall's **τ**) and significance between each Hexad user type and all others. N = 421. UT: User type; M: mean score; SD: standard deviation; Var: Variance; α: Cronbach's Alpha; A: Achiever; D: Disruptor; F: Free Spirit; P: Philanthropist; R: Player; S: Socialiser. ** Correlation is significant at the 0.01 level (2-tailed). * Correlation is significant at the 0.05 level (2-tailed).
